# The role of maternal age, education, birth experience and self-esteem in shaping anxiety before first postpartum intercourse

**DOI:** 10.3389/fpubh.2025.1708248

**Published:** 2025-12-11

**Authors:** Wiktoria Rozmarynowska, Agnieszka Czerwińska-Osipiak, Aleksandra Krawczyk, Anna Weronika Szablewska

**Affiliations:** Division of Obstetric and Gynaecological Nursing, Faculty of Health Sciences with the Institute of Maritime and Tropical Medicine, Medical University of Gdańsk, Gdańsk, Poland

**Keywords:** sexual activity, self-esteem, sexual activity after childbirth, sexuality, postpartum

## Abstract

**Introduction:**

The postpartum period involves significant physical and psychological changes that can impact a woman's sexual health. Anxiety experienced prior to resuming sexual intercourse after childbirth is a common yet understudied issue that may affect relationship satisfaction and overall wellbeing. While multiple biopsychosocial factors likely contribute to this anxiety, their interactions and particularly the role of self-esteem remain insufficiently understood. The aim of this study is to identify factors associated with anxiety before first postpartum sexual intercourse and to examine the relationship between self-esteem and sexual activity resumption among Polish women.

**Methods:**

We conducted an observational, cross-sectional study among 350 Polish women up to 12 months postpartum. Data were collected via an anonymous online questionnaire, which included socio-demographic characteristics, delivery details, sexual health aspects and self-esteem assessed using the Rosenberg Self-Esteem Scale (RSES). Logistic regression analysis was applied to identify significant predictors of postpartum sexual anxiety.

**Results:**

Higher maternal age (OR = 0.93) and more frequent previous sexual activity (OR = 0.78) were associated with reduced odds of anxiety. Conversely, higher education level (OR = 1.55), more time elapsed since delivery (OR = 1.14) and perceived delivery difficulty (OR = 1.31) significantly increased the risk of anxiety. Women with higher self-esteem reported more frequent sexual activity and lower anxiety levels. The final model showed moderate predictive accuracy (AUC = 0.65–0.70) and explained 7%−12% of variance.

**Discussion:**

Multiple factors, including maternal age, education, delivery experience and self-esteem level, are associated with anxiety prior to first postpartum intercourse. The findings highlight the importance of comprehensive perinatal care aimed at both physical recovery and psychological wellbeing. Healthcare providers should incorporate assessment of sexual health concerns and self-esteem issues into routine postpartum care to better support women during this transition.

## 1 Introduction

Sexual activity constitutes an integral component of human life and personality development. Women's body image tends to be more materially oriented compared to men's, which significantly influences the self-esteem of most women. The perinatal period, encompassing pregnancy, childbirth and the postpartum phase, represents a critical transition in women's perceptions of their body and sexuality. This period affects the form and nature of intimate contact experiences. Satisfaction with marital or partnership relations, as well as sexual satisfaction, has a fundamental impact on partners' determination of relationship stability and overall life satisfaction (1–3).

Notably, self-esteem, often defined as self-worth, significantly influences quality of life by affecting sexual activity and satisfaction derived from it, as well as the perception of one's relationship with a partner (4). Recent longitudinal research conducted in Ireland revealed concerning data regarding women's sexual health during the postpartum period. Approximately 50% of the surveyed women reported sexual difficulties 6 months after childbirth. Moreover, these problems not only persisted but actually intensified 1 year postpartum. The most commonly reported difficulty was decreased libido, which affected 46.3% of women at 6 months postpartum and persisted in 39.8% of participants after 1 year (5, 6). According to the literature, fewer than 10% of women resume sexual intercourse between seven and 31 days postpartum (7).

The resumption of sexual intercourse during the postpartum period requires partners to undergo a process of readjustment. This process is influenced by three main groups of factors: biological, psychological and socio-cultural. However, the relative importance and interplay of these factors, particularly in the context of anticipatory anxiety before the first postpartum intercourse, remain poorly understood. Moreover, the role of maternal self-esteem in this process has received limited attention. Among the biological aspects, factors such as lactation and perineal trauma can be identified, while psychological factors include stress and fatigue levels, as well as altered perception of body image. The social context is associated with the status of the partnership relationship. Partner support and effective communication within relationships may function as protective mechanisms against postpartum sexual problems. Women who sustain sexual activity typically display better social adjustment and improved overall wellbeing. Additionally, health problems occurring during the maternal period play a significant role. This multiplicity of interacting elements demonstrates that female sexuality constitutes an extremely complex phenomenon (3, 8, 9).

The perinatal period markedly influences female sexual functioning, reflected in reduced FSFI (Female Sexual Function Index) assessments. Sexual dysfunction rates among women increase by a factor of five post-delivery, with prevalence potentially reaching 40% in the postpartum population (10). In a literature review, progressive reduction was demonstrated in sexual activity frequency during gestation, with the most pronounced decrease occurring in the final trimester. Sexual behavior typically recommenced approximately 6–8 weeks postpartum, with complete restoration occurring only after a 6-month period. Concurrent alterations in sexual function were observed, including diminished orgasmic response, libido and sexual satisfaction, alongside increased dyspareunia. These modifications are attributable to multiple factors encompassing physical, psychological and social elements, concerns regarding potential adverse effects of sexual intercourse, insufficient or absent professional guidance on sexuality, delivery method and lactation practices (3, 6, 9, 11–14).

The relative significance of these multifaceted factors in shaping anticipatory anxiety before first postpartum intercourse remains inadequately explored. This cross-sectional study was therefore designed to quantify anxiety levels prior to first postpartum sexual intercourse among Polish women and to identify key associated socio-demographic, obstetric and psychological factors, with particular attention to the role of self-esteem. Simultaneous analysis of these factors provides a more comprehensive model for predicting postpartum sexual anxiety.

We hypothesized that multiple biopsychosocial factors, including maternal age, education level, delivery experience, and self-esteem, would be significantly associated with anxiety before first postpartum intercourse. Additionally, we expected that higher self-esteem would serve as a protective factor, reducing both the likelihood and intensity of postpartum sexual anxiety.

Healthcare practitioners can provide valuable assistance through enhanced education and support for expectant couples and new parents concerning sexuality, while adequately informing them about typical variations in sexual activity, interest, desire and responsiveness throughout the gestational period and postpartum phase (15). To facilitate a secure and adaptive parental transition, couple intimacy and sexual wellbeing may be strengthened through the promotion of behaviors including transparent communication, empathetic understanding and mutual support mechanisms. Protecting sexual health during the perinatal period should constitute an integral component of comprehensive perinatal healthcare management. Intimate connection represents an essential element in sustaining positive relational dynamics. This framework encompasses the understanding of sexual wellness while simultaneously promoting the health and overall wellbeing of a partnership, offspring, family unit and broader community (3, 16).

## 2 Material and methods

### 2.1 Study design and participants

This observational, cross-sectional study was conducted among Polish women in the postpartum period (up to 12 months after delivery). The main study objectives were to assess self-esteem levels using the Rosenberg Self-Esteem Scale (RSES) and to identify factors associated with resumption of sexual activity after childbirth.

Data were collected cross-sectionally using Computer-Assisted Web Interview (CAWI) methodology. Participants were recruited through nationwide online platforms and social media groups for mothers (total reach of approximately 15,000 members), excluding private and local groups to minimize selection bias.

The final sample comprised 350 women who met inclusion criteria: having a permanent partner both during pregnancy and the postpartum period, and being within one-12 months postpartum. Sample size was determined *a priori* via statistical power analysis using G^*^Power software (α = 0.05, power = 80%, effect size *r* = 0.3), indicating a minimum requirement of 176 participants. This effect size was selected based on similar research in postpartum sexual health reporting correlations of *r* = 0.25–0.35 between psychological factors and sexual outcomes (17), aligning with Cohen's convention for moderate effects in behavioral research (18). The final sample of 350 participants provided 97.7% statistical power for detecting the anticipated effect.

We examined whether maternal age, education level, time since delivery, perceived delivery difficulty, and previous sexual activity frequency were associated with anxiety before first postpartum intercourse.

The study is in adherence with STROBE guidelines and ethical approval was obtained from the Bioethics Commission of Medical University of Gdańsk (NKBBN/1017/2021-2022). All participants provided informed consent. All procedures followed the principles of the 1964 Declaration of Helsinki. Data quality was ensured through control questions and removal of duplicate IP submissions.

### 2.2 Data collection tools

An original, anonymous questionnaire was developed by a team of 10 expert midwives based on their clinical experience. The instrument contained 24 questions organized into two main sections: (I) socio-demographic and delivery characteristics (eight questions), and (II) postpartum experiences including feelings about delivery, sexual activity, anxiety, self-perception, and healthcare support (16 questions). Anxiety and stress before first sexual intercourse after childbirth were measured as follows: in a closed-ended question, respondents indicated whether they experienced anxiety before their first sexual intercourse after childbirth. If they answered affirmatively, in the subsequent question, they could indicate the intensity level of anxiety on a five-point scale (slider-type question). Questionnaire validity was ensured through expert evaluation using a five-point scale, with only items scoring ≥3 retained in the final version.

The third section, consisting of 10 diagnostic statements, included the Rosenberg Self-Esteem Scale questionnaire in its Polish version, examining the general level of self-esteem. This scale allows for the measurement of the general level of self-esteem, which, as revealed in self-description, can be treated as a stable trait rather than a momentary state. It is a useful tool, considering its simplicity and ease of use. Each respondent was required to indicate, on a four-point scale, the degree to which she agreed or disagreed with the statement. The mean score of the Self-Esteem Scale in the Polish adaptation is 29.49 (SD = 4.29) and it is lower than the average collected from all 53 countries (M = 30.85). For women in Poland, the mean score is 29.19 (SD = 4.28), thus achieving a lower level of self-esteem compared to Polish men (M = 29.94 with SD = 4.26). The normal distribution of scores is often disrupted by results below and above the mean. The results for Polish women below the mean are: 26, 27, 28, and above the mean: 30 (19).

The scale uses a four-point Likert scale format (1 = “strongly disagree,” 4 = “strongly agree”), with scores ranging from 10 to 40, where higher scores indicate higher self-esteem. The RSES demonstrates excellent psychometric properties, with Cronbach's alpha typically ranging from 0.77 to 0.88 across diverse populations.

### 2.3 Variables

The dependent variable was anxiety before first sexual intercourse after childbirth, measured as a binary outcome. For women who reported anxiety, its intensity was further assessed on a five-point ordinal scale.

Independent variables included maternal age, level of education, time elapsed since last delivery, subjective assessment of the last delivery (on a five-point scale, ranging from “very easy” to “very difficult”), and frequency of sexual intercourse with one's partner after childbirth.

The level of self-esteem, as measured by the RSES, was analyzed as a correlate to examine its associations with the other variables.

### 2.4 Statistical analysis

All statistical calculations were performed using the IBM SPSS 23 statistical package and Excel 2016 spreadsheet application. Qualitative variables were presented using frequencies and percentage values, while quantitative variables were characterized using arithmetic means and standard deviations. Statistical analysis was conducted via appropriate tests based on data distribution and variable characteristics. Spearman's rank correlation analysis was implemented to examine associations between continuous variables with non-parametric distribution. Logistic regression modeling was carried out to identify significant predictors, including both univariate and multivariate analyses. The adequacy of the logistic regression model was assessed using the Hosmer–Lemeshow goodness-of-fit test. The internal consistency of the Socioeconomic Status scale questionnaire was assessed by calculating Cronbach's alpha reliability coefficient. In all calculations, the significance level was set at *p* ≤ 0.05.

Additionally, descriptive statistics were used to summarize the data, including means, medians and standard deviations. Bivariate analyses (correlations), multivariate analyses (logistic regression) and odds ratios (OR) were also employed.

For statistically non-significant variables, the confidence interval for the Odds Ratio contains one, meaning these variables neither increase nor decrease the odds of the studied outcome. Therefore, the obtained ratio cannot be interpreted in the same way as for statistically significant variables.

## 3 Results

### 3.1 Study group characteristics

Based on multiple correspondence analysis, the profile of women participating in the study was determined. The typical woman participating in the study has given birth once and experiences anxiety/stress before first intercourse after delivery. She has higher education, is satisfied with herself, and disagrees that things are not going well for her. She believes she can do various things as well as most other people. She copes well with childcare. During pregnancy, she did not maintain regular sexual contact, and after delivery, she did not consciously postpone first sexual intercourse. This woman believes she possesses many positive traits and that she is a valuable person to the same degree as others, and disagrees that she does not have too many reasons to be proud of herself.

Detailed demographic and last delivery characteristics of the study population are presented in Table 1.

**Table 1:** Demographic data and information about the last delivery.

**Characteristics of the study group**	**No**.	**%**
Respondents	350	100
**Age**		
≤ 25	94	26.9
26–34	236	67.4
35–40	20	5.7
**Education level**		
Lower secondary (e.g., completed junior high school)	2	0.6
Vocational	11	3.1
Secondary	117	33.4
Higher (university degree)	220	62.9
**How much time has passed since your last childbirth?**		
About 1 month	1	0.3
Up to 2 months	15	4.3
Up to 3 months	77	22.0
Up to 4 months	47	13.4
Up to 5 months	4	1.1
Up to 6 months	51	14.6
Up to 7 months	71	20.3
Up to 8 months	61	17.4
Up to 9 months	19	5.4
Up to 11 months	1	0.3
Up to 12 months	3	0.9
**How would you rate your last delivery?**		
1—very easy	46	13.1
2—easy	74	21.2
3—neither difficult nor easy	105	30.0
4—difficult	79	22.6
5—very difficult	46	13.1
**How often do you have sexual intercourse with your partner**		
**(after childbirth)?**		
Daily	8	2.3
2–3 times a week	80	22.9
Once a week	91	26.0
Once every 2 weeks	60	17.1
Once a month	42	12.0
Less often than once a month	33	9.4
I have not had intercourse with my partner yet	36	10.3

The final stage of the survey involved completing the Morris Rosenberg Self-Esteem Scale—in the Polish version. The task for participants was to honestly indicate how much they agree (or disagree) with each of the 10 statements. Each statement is of diagnostic nature. Each response is given on a 4-point scale which is shown in Table 2.

**Table 2:** Results of SES-PL scale questionnaire.

**Descriptive statistics**	***N***	**Min**	**Max**	**M**	**SD**
I consider myself to be at least as worthy as others	350	1	4	1.66	0.65
I feel that I have a number of good qualities	350	1	3	1.70	0.60
Generally speaking, I tend to think that things are not going well for me	350	1	4	3.14	0.63
I am able to do things as well as most other people	350	1	4	1.83	0.62
I feel I do not have much to be proud of	350	1	4	2.99	0.72
I assume a positive attitude toward myself	350	1	4	2.05	0.67
On the whole, I am satisfied with myself	350	1	4	2.08	0.68
I wish I could have more respect for myself	350	1	4	2.49	0.88
All in all, I am inclined to think that I am a failure	350	1	4	2.62	0.87
I certainly feel useless at times	350	1	4	2.59	0.88

### 3.2 Outcomes

#### 3.2.1 Predictors of anxiety before first postpartum intercourse

Based on model comparison analysis, from a statistical perspective, the optimal model examining the effect of the studied variables on anxiety/stress before first sexual intercourse after childbirth is one containing five independent variables and an intercept.

The model's goodness of fit is relatively low (*R*^2^Pseudo = 0.07, *R*^2^Nagelkerke = 0.12 and *R*^2^Cox-Snell = 0.09). However, the model is statistically significant (*p* = 0.000008 for the likelihood ratio test), indicating that the independent variables included in the model are statistically significant. The Hosmer–Lemeshow test result indicates non-significance (*p* = 0.33). It should be noted that in the case of the Hosmer–Lemeshow test, non-significance is desirable as it points to similarity between observed frequencies and predicted probabilities. The logistic regression model in the study group (*n* = 350) for the analyzed variables is presented in Table 3.

**Table 3:** Model for analyzed variables.

**Variable**	***b***	**b error**	**−95% CI**	**+95% CI**	**stat. Walda**	***p*-Value**	**Odds Ratio**	**−95% CI2**	**+95% CI3**
W. free	1.179567	1.049086	−0.876603	3.235737	1.264221	0.260854	3.252965	0.416194	25.425099
Age	−0.076028	0.03399	−0.142647	−0.00941	5.003347	0.025298	0.92679	0.86706	0.990634
Education	0.440006	0.223462	0.002029	0.877984	3.87713	0.048948	1.552717	1.002031	2.406044
Time since delivery	0.13144	0.053878	0.025842	0.237039	5.951655	0.014703	1.14047	1.026179	1.26749
Perceived delivery difficulty	0.27133	0.101807	0.071792	0.470868	7.102973	0.007696	1.311708	1.074432	1.601383
Previous sexual activity frequency	−0.242371	0.075521	−0.39039	−0.094353	10.299724	0.001331	0.784765	0.676793	0.909962

The odds of a woman experiencing anxiety/stress before first postpartum sexual intercourse depend on the following variables, as described by odds ratios:

- Age [OR = 0.93 (95% CI: 0.88–0.99)]: the older the woman, the lower the odds of experiencing anxiety/stress before first postpartum sexual intercourse. Each additional year of age reduces the odds by 7%.
- Level of education [OR = 1.55 (95% CI: 1.00–2.41)]: the higher the education level a woman has, the greater the odds of experiencing anxiety/stress before first postpartum sexual intercourse. Higher education increases the odds by 55%.
- Time since delivery [OR = 1.14 (95% CI: 1.03–1.27)]: the longer the time that has elapsed since the last delivery, the greater the odds of experiencing anxiety/stress before first postpartum sexual intercourse. Each additional time unit increases the odds by 14%.
- Assessment of last delivery [OR = 1.31 (95% CI: 1.07–1.60)]: the more difficult a woman rates her last delivery, the greater the odds of experiencing anxiety/stress before first postpartum sexual intercourse. Each increase in difficulty rating increases the odds by 31%.
- Frequency of sexual intercourse after previous deliveries [OR = 0.78 (95% CI: 0.67–0.91)]: the more frequently a woman had sexual intercourse after previous deliveries, the lower the odds of experiencing anxiety/stress before first postpartum sexual intercourse. Higher frequency reduces the odds by 22%.

**Figure 1:**
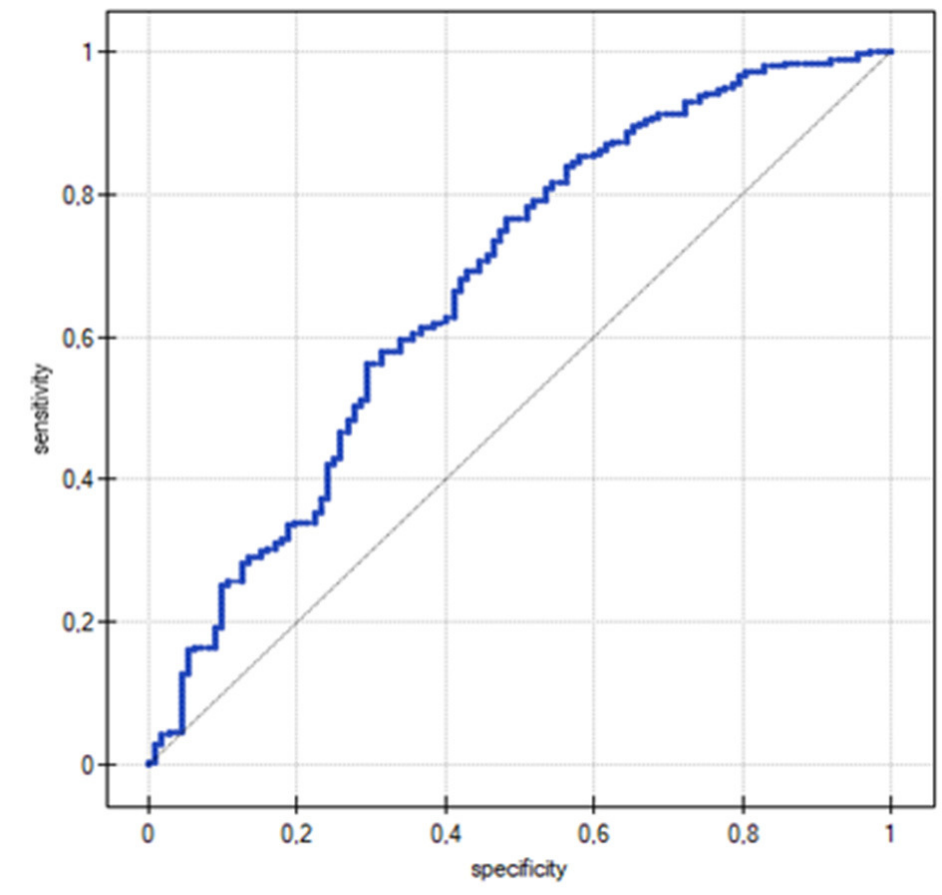
ROC curve.

The discriminatory ability of the logistic regression model was assessed using ROC curve analysis, which is presented in Figure 1. The analysis revealed that the five-variable model achieved fair to good discriminative ability (AUC ≈ 0.65–0.70) in distinguishing between women who experienced postpartum sexual anxiety and those who did not. The ROC curve demonstrated moderate discriminative performance, positioned well above the diagonal line of no discrimination, indicating that the model performs significantly better than random chance (*p* = 0.000008).

The ROC curve performance aligns with the model's statistical characteristics. Despite relatively low pseudo-*R*^2^ values (*R*^2^Pseudo = 0.07, *R*^2^Nagelkerke = 0.12, *R*^2^Cox-Snell = 0.09), the curve demonstrates meaningful discriminative capacity. The satisfactory Hosmer–Lemeshow test result (*p* = 0.33) further supports the model's calibration, indicating good agreement between predicted probabilities and observed frequencies.

The analysis suggests that the developed model has practical clinical utility as a screening tool for identifying women at an elevated risk of experiencing anxiety before resuming sexual activity. While the predictive performance is rather moderate than excellent, this level of discrimination is clinically meaningful for a complex psychological outcome influenced by multiple biopsychosocial factors. The model's ability to differentiate between high- and low-risk women supports its potential application in clinical practice for targeted interventions and counseling approaches.

A relationship between the Rosenberg Self-Esteem Scale and the assessment of childbirth as well as anxiety about first sexual intercourse after childbirth was described. A detailed description of the relationship is presented in Table 4.

**Table 4:** SES and assessment of delivery and anxiety before first intercourse after childbirth.

**SES**	***N***	**rHO**	***p-*Value**
Assessment of delivery (1 = very easy - 5 = very difficult)	350	−0.16	0.003
Anxiety before first intercourse (1 = low-level stress - 5 = extreme stress)	278	−0.12	0.037

## 4 Discussion

This cross-sectional observational study was conducted to evaluate self-esteem levels and sexual activity patterns among Polish women during the postpartum period, focusing on factors influencing anxiety/stress before resuming sexual intercourse after childbirth.

In this analysis, we identified several significant predictors of postpartum sexual anxiety. Increasing age was associated with reduced odds of experiencing anxiety before first postpartum sexual intercourse, supported by Zyśk and Durka, who observed that older women reported higher self-esteem and more frequent feelings of attractiveness (20). Interestingly, in their study, it was also shown that younger women more frequently reported higher sexual satisfaction after childbirth, suggesting a divergence between emotional readiness and physical fulfillment. This contrast may reflect the assumption that older women possess greater emotional resilience and body acceptance, while younger women may experience more intense physical satisfaction or fewer physiological barriers to intercourse.

Contrary to expectations, higher education increased anxiety odds [OR = 1.55 (95% CI: 1.00–2.41)]. Potkonjak et al. indicated that women typically become pregnant upon completing their education, creating a correlation between higher educational attainment and advanced maternal age. It was further shown that women with lower education levels demonstrate reluctance to participate in sexual health research due to cultural prejudices (21). Van der Zee-van den Berg et al. reached similar findings, suggesting higher educational level was associated with increased general postpartum anxiety (22). The elevated anxiety among more educated women may result from greater exposure to health information, analytical thinking patterns promoting systematic worry and higher performance expectations.

This finding challenges the assumption that education universally serves as a protective health factor. While higher education provides better access to healthcare information, it may paradoxically increase cognitive load through greater awareness of potential complications, more analytical processing of bodily changes, and heightened self-monitoring, suggesting that highly educated women may require tailored support addressing their specific information-processing styles.

Time since delivery emerged as another significant predictor, with each additional time unit increasing anxiety odds by 14%. This finding contrasts with Artieta-Pinedo et al., who reported that women resuming sexual relations were more likely to experience body image dissatisfaction than those remaining sexually inactive (23).

Perceived delivery difficulty significantly increased anxiety odds by 31% [OR = 1.31 (95% CI: 1.07–1.60)]. Handelzalts et al. highlighted that subjective childbirth experience significantly influences postpartum sexual satisfaction, with negative experiences leading to reduced sexual desire and increased anxiety (24). In the MOODS study by de Sousa et al. it was confirmed that operative vaginal deliveries were associated with lower postpartum sexual function, particularly with regard to pain, arousal and satisfaction (25). Barbara et al. found that women who underwent cesarean sections reported better sexual outcomes compared to those who had instrumental vaginal deliveries, noting that even spontaneous vaginal births associated with somewhat diminished orgasm quality, suggesting birth-related trauma can impair postpartum sexual wellbeing (26, 27). Faisal-Cury et al. concluded that cesarean section was not consistently protective, but instrumental vaginal delivery significantly increased sexual dysfunction risk (28–30). Krasucki and Barczuk showed that childbirth-related anxiety acts as a mediator between delivery type and sexual satisfaction (31).

Fear of childbirth (FOC) during pregnancy represents an important psychological factor that may contribute to postpartum sexual anxiety. Previous research has demonstrated that high antenatal FOC is associated with increased risk of perinatal anxiety, depressive symptoms, and post-traumatic stress after childbirth (32, 33). Moreover, concerns related to pain, bodily damage, and changes in body image can extend into the postpartum period, influencing women's sexual self-perception and readiness to resume intercourse (34). Although our study did not include a direct antenatal measure of FOC, women's subjective perception of delivery difficulty was significantly associated with their fear before first postpartum intercourse, suggesting that a stressful or traumatic birth experience may mediate this relationship. Future studies should incorporate standardized antenatal assessments [e.g., Wijma Delivery Expectancy Questionnaire (W-DEQ) or Fear of Birth Scale (FOBS)] to examine whether fear of childbirth predicts postpartum sexual anxiety through pathways such as traumatic birth experience or diminished self-efficacy (35).

A crucial finding was the relationship between self-esteem and sexual frequency. Women engaging in daily sexual intercourse exhibited significantly higher self-esteem compared to those with activity less than once per month. This aligns with the findings of Sipiński et al., who emphasized that self-esteem doubts are linked to reduced sexual desire and activity, and frequent changes in physical appearance as well as previous pregnancies significantly influence the frequency of sexual activity (36).

Filipek and Slizień-Kuczapska reported that 65 and 75% of women, respectively, did not experience satisfaction upon resuming postpartum sexual activity (37, 38). Our findings confirmed this strong link between self-esteem and sexual frequency, highlighting how pain, anxiety, body image changes, shame and lack of perceived attractiveness can diminish both the frequency and quality of postpartum sexual experiences.

Previous sexual experience showed protective effects, with higher frequency of intercourse after prior deliveries reducing anxiety odds by approximately 22%. As emphasized by Makara-Studzińska et al. and Lund et al., sexuality remains a deeply significant aspect of identity during the transition to motherhood, with pregnancy experience, adaptation to maternal role and evolving perceptions of sexual attractiveness all contributing to postpartum sexual wellbeing and long-term sexual self-esteem (39, 40).

Our results demonstrate consistency with research emphasizing the multidimensional nature of postpartum sexual health. The findings align with current literature underlining both the physical and psychological dimensions of childbirth impact on sexual function. Together, these findings affirm that perceived delivery difficulty—through its link to pain, trauma, anxiety, and psychological readiness—is a key predictor of postpartum sexual stress. Information in the existing literature confirms that continuous support for a woman throughout the entire puerperium is a crucial factor contributing to a woman's overall wellbeing (41, 42).

These findings underscore the need for comprehensive postpartum care addressing both physical and emotional aspects of sexual health (43). Filipek et al. drew attention to the widely held belief that women should abstain from intercourse for 6 weeks postpartum—often treated as a dogma rather than individualized medical advice. Many women resumed activity due to fear of partner impatience or guilt over prolonged abstinence rather than personal readiness (37).

The existing literature confirms that continuous support throughout the entire puerperium is crucial for overall wellbeing. Acele et al. highlighted healthcare professionals' crucial role in emphasizing women's sexual health during pregnancy, childbirth and the postpartum period, noting that lack of communication, guidance and preparation for postpartum intimacy can increase stress and anxiety among new mothers (17). Sipiński et al. stressed that insufficient emotional support and neglect of sexual health by medical staff represent serious oversights, which can negatively impact couples' sexual relationships (36).

Healthcare providers should recognize that higher education does not reduce postpartum sexual concerns and should employ culturally sensitive approaches across all educational levels. The predictive model showed moderate ability to identify high-risk women, suggesting these findings can guide screening and support strategies (44). Proactive education, reassurance and open conversations about intimacy could reduce distress and support healthy postpartum sexual adjustment (45). Beyond identifying risk factors, our findings point to the preventive potential of evidence-based antenatal interventions. Recent research, such as the randomized controlled trial by Motz demonstrates that structured prenatal programs—including hypnosis-based and mindfulness-based approaches can significantly reduce perinatal anxiety and stress. Integrating such interventions into routine care could build psychological resilience, reframe birth-related expectations, and provide coping tools that may directly mitigate the anxiety associated with resuming postpartum sexual activity. This represents a promising, actionable pathway from research to clinical practice for improving postpartum sexual wellbeing (46).

In addition to the psychological and demographic factors examined, other determinants such as relationship satisfaction, partner support, and physical recovery may significantly influence postpartum sexual anxiety. Recent research shows that perceived social support and marital satisfaction mediate postpartum stress (47) and that partner support and relationship quality affect women's birth experience and early wellbeing (48). Moreover, physical recovery factors—including perineal trauma and mode of delivery—have been shown to impair sexual function up to 24 months postpartum (49). Although these variables were not measured in our study, they likely act as unmeasured covariates and should be included in future work to develop a more comprehensive explanatory model of postpartum sexual outcomes.

Future research should address several limitations of the current study. Longitudinal designs are needed to establish causal relationships between identified predictors and postpartum sexual anxiety. Studies should incorporate standardized antenatal assessments of childbirth-related fear, such as the Wijma Delivery Expectancy Questionnaire (W-DEQ) or Fear of Birth Scale (FOBS), to examine whether fear of childbirth predicts postpartum sexual anxiety through potential mediating pathways such as traumatic birth experiences or diminished maternal self-efficacy. Including variables such as relationship quality, mental health history, and social support would strengthen the model, while multicenter recruitment would enhance generalizability across diverse populations and healthcare settings.

Our findings allow to directly identify the key determinants of postpartum sexual anxiety. A more difficult birth experience significantly increased anxiety risk, while higher maternal age and self-esteem served protective roles. Contrary to expectations, higher education was associated with greater anxiety, highlighting the complex nature of this issue.

### 4.1 Strengths and limitations

This study was carried out to identify key predictors of postpartum sexual anxiety, including age, education level, time since delivery, perceived delivery difficulty and previous sexual experience, with the predictive model demonstrating moderate ability to identify high-risk women for healthcare screening. The study also provides valuable insights into the under-researched population of Polish postpartum women. However, several limitations should be considered. The study relied on self-reported data susceptible to recall and social desirability bias and employed a cross-sectional design preventing causal inference. The model's relatively low explanatory power (pseudo-*R*^2^ values between 0.07 and 0.12) suggests that other significant factors not measured in this study, such as relationship quality, pre-existing mental health conditions, or level of social support, likely contribute to the experience of postpartum sexual anxiety. Future research should aim to incorporate these variables to build a more comprehensive predictive model. Furthermore, the generalizability of our findings may be limited by the exclusive focus on Polish women and the online recruitment strategy, which may not fully represent the entire postpartum population, particularly women with lower socioeconomic status or limited internet access. Despite these limitations, the findings emphasize the need for comprehensive postpartum care, addressing both the physical and emotional aspects of sexual health recovery.

## 5 Conclusions

In conclusion, our findings challenge the assumption that higher educational attainment protects against postpartum sexual health concerns. The unexpected association between higher education and increased anxiety risk underscores the need for healthcare providers to adopt a universal, biopsychosocial approach to postpartum care that addresses psychological vulnerability across all socioeconomic groups. Clinical practice should recognize that educated women may be at increased risk for anxiety due to higher performance expectations, greater health awareness, or analytical thinking patterns, and provide tailored support accordingly. The study findings have important consequences for postpartum care organization. Clinical practice should incorporate routine screening for sexual anxiety during follow-up visits, particularly for highly educated women and those with traumatic birth experiences. Medical personnel require training in identifying risk factors and therapeutic communication, necessitating standardized assessment tools that extend beyond traditional 6-week recommendations.

At the system level, specialized sexual health clinics and interdisciplinary teams including psychologists, physiotherapists and sexologists should be integrated into standard postpartum care. Early intervention protocols within the first weeks after delivery are crucial as waiting for spontaneous improvement may worsen problems, which was clearly demonstrated in our results. Health education should include materials tailored to patients' educational levels and support groups for women after traumatic births. Implementation of programs for building self-esteem and positive body image is essential, given the strong relationship between self-esteem and sexual functioning. These initiatives require further prospective studies evaluating intervention effectiveness as well as the development of diagnostic tools specific to postpartum sexual anxiety.

## Data Availability

The raw data supporting the conclusions of this article will be made available by the authors, without undue reservation.
